# Profile and outcome of patients with post-neonatal tetanus in a tertiary centre in south west Nigeria: any remarkable reduction in the scourge?

**DOI:** 10.11604/pamj.2015.21.254.6488

**Published:** 2015-08-06

**Authors:** Barakat Adeola Animasahun, Olusegun Henry Gbelee, Aminat Titilayo Ogunlana, Olisamedua Fidelis Njokanma, Olumuyiwa Odusanya

**Affiliations:** 1 Department of Paediatrics and Child Health, Lagos State University College of Medicine, Ikeja, Lagos, Nigeria; 2 Department of Paediatrics, Lagos State University Teaching Hospital, Lagos, Nigeria; 3 Department of Community Health, Lagos State University College of Medicine, Ikeja, Lagos, Nigeria

**Keywords:** Tetanus, immunization, developing, lower limb, socio-economic class

## Abstract

**Introduction:**

The incidence of tetanus has remained unacceptably high in developing countries. We aimed to describe the profile and outcome of children with tetanus admitted at the Lagos State University Teaching Hospital (LASUTH), Ikeja.

**Methods:**

A prospective and cross-sectional study of children aged 1 month to 12 years of age admitted with clinical diagnosis of tetanus, between January 2011 and December 2013, at the Paediatric department of LASUTH. The age, sex, presenting complaint, immunization status, portal of entry, socio-economic class, complications, duration of admission and outcome of the subjects were analyzed using Microsoft Excel supplemented with Statistical Package for Social Sciences (SPSS) version 17.0. Level of significance set at p< 0.05.

**Results:**

A total of 49 subject participated in the study. Male: Female ratio was 1.7: 1.0. mean age ± SD of 6.5± 3.2 years. Only 24.5% of the subjects were fully immunized, lower limb injury was the most common portal of entry (34.7%). Majority (79.6) were of the middle and lower social classes. Most of the subjects (67.3%) presented with generalised spasm. Only 1 patient (2.0%) did not have trismus. Case fatality rate was 4.1%.

**Conclusion:**

Tetanus is still prevalent among children in our environment. It is commoner among those with no immunization or incomplete immunization, commoner in those in the middle and lower social class. Lower limb injury was the most common portal of entry. Trismus was a common presenting feature. There is a need to develop programmes with will help improve compliance to immunization.

## Introduction

Tetanus remains a non-communicable disease of major public health concern especially in developing countries [1] such as Nigeria. The incidence of tetanus has remained unacceptably high in developing countries although tetanus is a vaccine-preventable disease [2, 3]. Although the extended programme of immunization has been in existence for over three decades [4], the persistent high incidence of tetanus has continued to compete with the success of the programme [4–6]. The contributory factors to this continued high incidence of tetanus include lack of, or incomplete immunization among other reasons.2 Increasing incidence of tetanus infection over the last two decades have been described in Hospital-based studies in Nigeria and this has been attributed to lack of sustenance of the immunization programme among other reasons [5, 7, 8]. Mortality rates have also remained high both in adults and children in Nigeria and parts of Africa [9–14]. Tetanus is reported to account for 3.7% of childhood deaths among hospitalized children in Nigeria with a case fatality rate of 18% while hospital based studies of adult population in Nigeria has shown an unacceptably high mortality rate of 56.2% [1] to 64% [9, 10]. A rate of 95% immunization coverage is necessary for the sustained control of vaccine-preventable diseases [15]. The reported DPT3 coverage rate initially showed remarkable improvement during the first decade after launching the EPI from 31% to about 80.8%. This was followed by an intervening period of steady decline to about 25% before another improvement was again recorded [2, 16, 17]. Furthermore, widespread disparities in the coverage of immunization programme persist between and within rural and urban areas, regions and communities in Nigeria [18]. The non-attainment of 95% immunization coverage rate as well as the absence of booster doses of tetanus toxoid as part of childhood immunization necessitates a constant assessment of the profile and outcome of the patients with childhood tetanus. There has been no review of the incidence, morbidity and mortality of post-neonatal Tetanus in Nigeria in the last decade. Hence we aim is to describe the profile and outcome of children with tetanus admitted at the Lagos State University Teaching Hospital (LASUTH), Ikeja. This is to enable us proffer new strategies in reducing the incidence, morbidity and mortality associated with the illness.

## Methods

A review of the prospectively obtained records of all children from the age of 1 month to 12 years of age admitted with clinical diagnosis of tetanus between January 2011 and December 2013 in Paediatric department of LASUTH. Lagos State University Teaching Hospital is an urban tertiary center located in the heart of Lagos, Nigeria. It serves as a referral center for public and private hospitals in Lagos and her neighboring states. The age, sex, presenting complaint, immunization status, portal of entry, socio-economic class, complications, duration of admission and outcome of the subjects were analyzed. Subjects were classified into socio-economic classes (SEC) using the Oyedeji classification [19] which utilizes the educational background and occupation of parents. The socio-economic classes were further grouped into upper (classes I and II), middle (class III) and lower (classes IV and V) SEC. Inclusion criteria were; age older than one month up to 12 years old with a clinical diagnosis of tetanus. Immunization status was determined using the current National Program on Immunization (NPI) schedule. A child was said to be fully immunized for tetanus if he or she had received all of the three doses of Diphtheria, Pertussis and Tetanus (DPT) as at the time he or she was recruited for the study.

### Ethical clearance

Ethical clearance for the study was obtained from the Research and Ethics Committee of the Lagos State University Teaching Hospital. Informed consent was sought from parents or caregivers before enrollment in the study.

### Statistical analysis

Data analysis was done using Microsoft Excel statistical package supplemented with Statistical Package for Social Sciences (SPSS) version 17.0. Mean, standard deviation and other parameters were generated as necessary for continuous data. Means of continuous variables were compared using the Student t test and proportions using Chi-square test. Level of significance set at p< 0.05.

## Results

**Figure 1:**
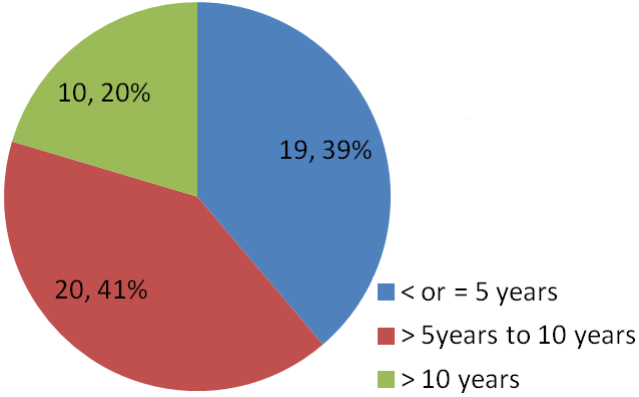
Age categories of respondents

**Figure 2:**
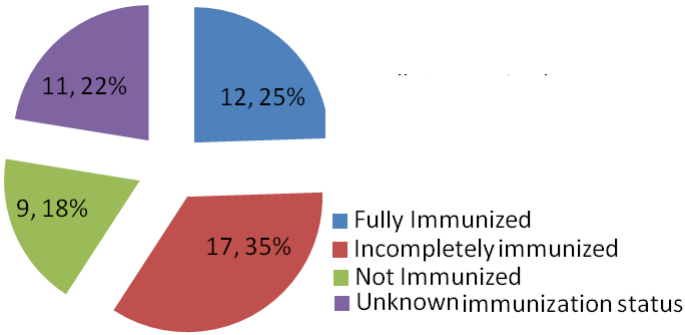
Immunization status of subjects

**Figure 3:**
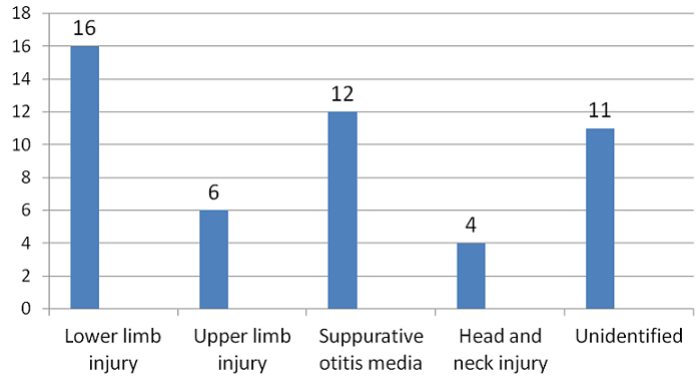
Portal of entry of the causative organism in subjects

**Figure 4:**
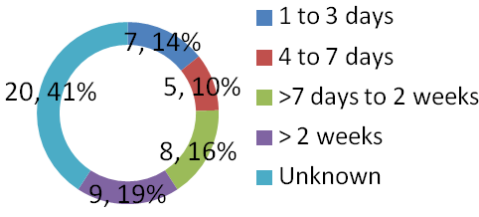
Incubation period of tetanus in subjects

**Figure 5:**
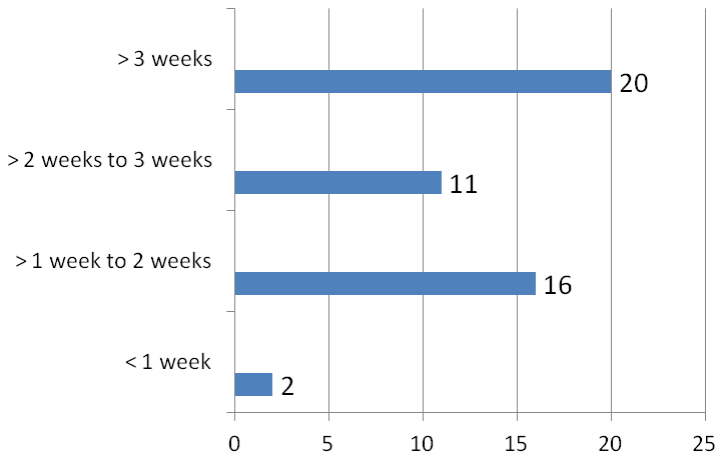
Duration of hospital stay of subjects

**Figure 6:**
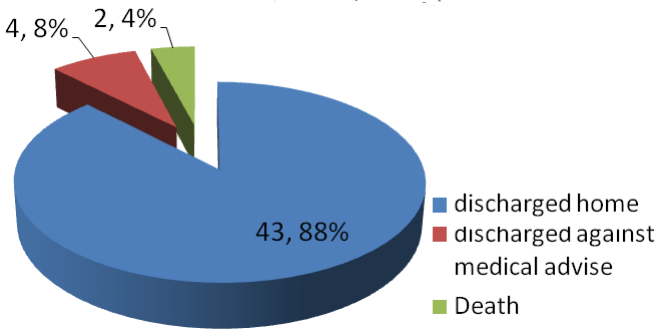
Outcome of subjects

A total of 8280 patients were admitted during the study period out of which 49 had tetanus. This represented 0.6% of the total admission in our children emergency room during the period under review. 17,20 and 12 subjects were admitted in 2011, 2012 and 2013 respectively which represented 0.6%, 0.8% and 0.4% of the total admissions for each year hence the prevalence of tetanus among children admitted in the children emergency department of LASUTH during the study period was 6 per 1000. There were 31 (63%) male giving a male: female of 1.7:1.0. The ages of the patients ranged between 1.5 years-12 years with a mean age ± SD of 6.5±3.16. The age distribution of the study subjects seen in Figure 1. Twelve subjects (24.5%) were fully immunized, 17 (34.7%) had incomplete immunization, 11(22.4%) subjects were never immunized while the immunization status of 9 (18.4%) subjects were unknown as shown in Figure 2. In terms of the portal of entry, Lower limb injury was the most common portal of entry in 17 (34.7%) as shown in Figure 3; Table 1 enumerates the socioeconomic status of the subjects. There was no subject in the social class I, 14 (28.6%) were in social class II, while 22 (44.9%) and 3(6.1%) were from social classes IV and V respectively. Data from nine subjects were incomplete thus their social class status could not be determined. There was no available data for the incubation period of most (20, 40.8%) of the patients as shown in Figure 4. In terms of the mode of presentation as shown in Table 2. Majority of the subjects (67.3%) had generalised tetanus. Twenty nine (59%) patients presented with both trismus and generalized spasm, neck pain and stiffness in 23 (44.9%) and fever in 13 (26.5%). Dysphagia, chest pain and inability to walk were less common modes of presentation. Only 1 patient (2.0%) did not have trismus as part of his presenting complaint. Table 3 shows the complications seen in the subject. Most of the patients (41,83.7%) had no complications during their hospital stay. Four (8.2%) patients developed aspiration pneumonitis, 2(4.1%) developed Acute kidney injury (AKI) while constipation and dark urine was recorded in one patient each. Duration of hospital stay and outcome are shown in Figure 5 and Figure 6 respectively. 41% of the subjects stayed for more than three weeks with a mean with standard deviation of 19.8 + 9.4. Case mortality rate was 4.1%.

**Table 1:** Socioeconomic distribution of subjects

Social class	number	Percentage
Upper	1	2.0
Middle	14	28.6
Lower	25	51
Unknown	9	18.4
Total	49	100

**Table 2:** Presenting features in subject

Presenting Features	Number	Percent
Generalised Spasm only	33	67
Generalised spasm and trismus	29	59
Neck pain and stiffness	23	44.9
Fever	13	26.5
+Others		

Some presented with more than one features

+ Includes dysphagia, chest pain and inability to walk which were less common modes of presentation.

**Table 3:** Complications in the subjects

Complications	Number	Percent
Aspiration pneumonitis	4	8.2
Acute Kidney Injury(AKI)	2	4.1
Constipation	1	2.0
Dark urine	1	2.0
None	41	83.7
Total	49	100

## Discussion

The study aimed to describe the pattern, clinical profile and outcome of children with tetanus at the Lagos State University Teaching Hospital, Ikeja, Lagos. In this study, 0.6% of the total number of children admitted in the study centre during the study period had tetanus. This proportion is high, in an urban area and commercial capital of the country where immunization is supposedly expectedly to be more available and accessible, the reason for this high proportion may still be explained by the deadly triad of poverty, ignorance and disease since one of the findings in the study showed that more than half of the subjects were froom the lower socio-economic classes which may suggest ignorance about prevention of tetanus by the parents. However the proportion of 0.6% found in this study is lower than 1.1% documented by Anah [10] in 2008 and 1.8% documented by Oyedeji [20] in 2012 (p= 0.3 and 0.02 respectively). The reason for the lower proportion found in this study may be explained by the fact that the current study centre is a more urban centre compared with the other two centres hence it may be plausible to assume that the parents living in the current study centre (Lagos) have better access to information on the need to immunize their children and also have a better access to the vaccines. The lower proportion of tetanus subjects among those admitted during the study period may also be a reflection of decreasing trend in reported cases which has been seen in some centres [21]. There were significantly higher number of males compared with females, similar findings were documented by earlier authors in Nigeria and ouside Nigeria [6, 7, 10, 21, 22]. This may be explained by the likelihood of the male child to be more adventurous and to play barefoot which puts them at greater risk of injuries (especially of the lower limbs) and subsequently tetanus infection. The mean age of 6.5 +3.2 years found in this study was similar to that documented by Alhaji [7] among children in the Northern part of Nigeria but lower than 5.83 + 3.4 documented by Akunhwa et al [22] and Tullu et al [21]. In all the reports most of the subjects were in the school age which suggests that the current programme on immunization confers immunization on of the child up to age of five years, this further emphasises the need to include booster doses of tetanus toxoid in all children as part of the National programme on Immunization. This study demonstrated that social class of parents plays an important role in the likelihood of having complete immunization; twenty-six (53%) of the subjects belong tothe lower social class and had either no or incomplete immunization. Similar finding were documented by Akuhwa et al [22] and Adegboye et al [23]. The reasons given by the caregivers of our patients for no or incomplete immunization included relocation from place of residence, mother's ill-health and lack of funds for transportation and payment at health centres. There has been wide disparities in the coverage of immunization programme between and within rural and urban areas, regions and communities in Nigeria [18]. The DPT3 completion rate of 24.5% was comparably lower than the estimated 1994 World Health Organization [24] coverage rate of 79% and 80.8% coverage rate reported by Odusanya et al [2] in 2008. Lower limb injury was the commonest portal of entry in our subjects. Similar findings were reported by earlier authors [7, 13, 25]. Most of our subjects were from the lower socio-economic class which makes it more likely that these children engage in predisposing acts to lower limb injury, such as walking bare foot. In the current study, there were no available data for the incubation period of most (19,38.8%) of the patients because parents and caregivers of those subjects could not ascertain the period of the injuries or infection that served as portal of entry. In the mode of presentation of the subjects, generalised spasm and trismus were the most prominent, Only 1 patient did not have trismus as part of his presenting complaint. Uncommon mode of presentation found in this study include dysphagia which is due to spasm of the larynx, chest pain and inability to walk are due to spasm and rigidity of the muscles [26]. Fever was the mode of presentation in about 13% of the subject. It may be due to intense output of energy, which accompanies tetanic seizures it may also be a manifestation of infection in them. A high fever at presentation is said to adversely affects prognosis [27]. This was the case in the current report as the two subjects who died presented with fever.

Prolonged hospitalization averaging 23-27 days reported by various workers [3, 21] is in agreement with the 20 days amongst our patients. Duration of admission of subjects in the current study ranged from 6-39 days. Majority (63.2%) of our patients stayed beyond two weeks on admission which is similar to the report by Alhaji et al [7]. This may be due to the number of days taken to achieve spasm control. Prolonged hospital stay places a huge burden on family and state resources and contributes significantly to school absenteeism. The duration of admission did not however influence the decision to discharge against medical advice as 50% each of the patients who discharged against medical advice stayed below and above 14 days on admission. Complications such as aspiration pneumonitis, Acute kidney Injury (AKI) constipation and dark urine wer reported in our study. Aspiration may be due to heavy sedation to suppress spasm in these subjects leading to suppression of protective airway reflexes and pooling of secretions (inspite of appropriate airway toilet), Acute kidney injury and passage of dark coloured urine may be due to myoglobinuria which results from excessive breakdown of RBC in the muscles due to muscle spasm, constipation is due to tight abdominal muscles experienced [21, 27]. Autonomic imbalances (sudden cardiac arrest, sympathetic overactivity, hypotension/hypertension, bradycardia/tachycardia), hypox-emia, septicemia, GI hemorrhage, paralytic ileus, urinary tract infections, vertebral fractures, decubitus ulcer and constipation have been reported as complications [21] but were not the case in our subjects. In terms of the outcome of the patients, The two mortalities occured on 6 days and 14 days of admission respectively. Both subjects were males, one had no immunization while the other had incomplete immunization. They both belong to the low socio-economic class and developed complications (AKI and aspiration pneumonitis respectively) before their demise. Tetanus is said to be a self-limiting disease, where if the patient can be kept alive for 3 weeks, can completely recover [26]. The case fatality rate of 4.1% was much lower than what was reported in previous studies [3, 10, 25] in teaching hospital settings in our locality. Possible reasons for this reduced mortality include improvement in overall care of tetanus patients over time with attendant improvement in provision of supports for these subjects [26]. The free health policy at the study centre which is available for children from birth to 12 years of age could have also contributed to the outcome of this patient as it encourages early presentation, and availability of supports in terms of man-power and medications for these subjects. The limitations of the study were incomplete documentation and lack of follow-up of patients who discharged against medical advice. Most of the patients and parents alike could also not remember the portal of entry.

## Conclusion

Poverty and ignorance play a significant role in the prevalence of childhood tetanus. This is more so because the coverage rate of immunization is less amongst children born into families of low socioeconomic class. Tetanus is commoner among those above five years of age. The scurge however appear to be reducing. We recommend that booster doses of tetanus toxoid should be made mandatory at school entry and repeated between 10 and 11 years of age.
